# Annotated whole-genome sequences of fermentative and spoilage associated Bacilli and proteobacteria autochthonous to commercial cucumber fermentation

**DOI:** 10.1128/mra.00926-23

**Published:** 2024-02-01

**Authors:** Clinton A. Page, Sheron Simpson, Ilenys M. Pérez-Díaz, Adam R. Rivers

**Affiliations:** 1 USDA Agricultural Research Service, Food Science and Market Quality and Handling Research Unit, Raleigh, North Carolina, USA; 2 USDA Agricultural Research Service, Genomics and Bioinformatics Research Unit, Stoneville, Mississippi, USA; University of Southern California, USA

**Keywords:** fermentation, food microbiology

## Abstract

We report 36 whole-genome sequences, along with annotations, of fermentative (*n* = 12) and spoilage associated (*n* = 6) lactic acid bacteria, *Lysinibacillus* (*n* = 3), *Streptococcus* (*n* = 1), and Proteobacteria (*n* = 14) isolated from commercial cucumber fermentations. Fifty-three percent of the genome sequence assemblies consist of 1–4 contigs, and the remainder have fewer than 16.

## ANNOUNCEMENT

We document genome sequences of multiple Bacilli and Proteobacteria isolated from commercial cucumber fermentation. Nine genome sequences are higher quality versions of previous submissions (1). Three genome sequences represent the nisin producer *Lactococcus lactis* (2) and putative bacteriocin producing *Pediococcus pentosaceus* and *Leuconostoc mesenteroides* strains (3). The remainder are Proteobacteria (*n* = 14) associated with fermented cucumber bloater defect (4) and Bacilli (*n* = 9) relevant to the instability of cucumber fermentation (5).

Table 1 describes the collection site of the isolates. Most fermentative lactic acid bacteria were collected, classified, and identified in surveys of commercial cucumber fermentations conducted between 2009 and 2010 as described by Pérez-Díaz et al. (6). LA0445 was derived from anaerobic fermentation of cucumbers (7). The isolations of *Lactococcus lactis* NCK401 (2) and *Pediococcus pentosaceus* LA0061 (previously identified as *P. cerevisiae* L-7230) (3) were conducted in Raleigh, NC, USA from cover brine samples derived from undefined sources. *Leuconostoc mesenteroides* FFL48 (previously identified as *P. cerevisiae* FBB-61) was collected and isolated in Marshall, MI, USA (8). The Proteobacteria and spoilage associated lactic acid bacteria included here were isolated as described by Pérez-Díaz et al. (6) and Pérez-Díaz et al. (4), respectively. *Lysinibacillus* strains were isolated from spoiled commercial cucumber fermentations in Mt. Olive, NC, USA as described by Medina et al. (5).

**TABLE 1:** Accession numbers and genome statistics for isolates described in this announcement^*a*^

Strain IDs	NCBI accession number	SRA accession number	Assembly size (bp)	Contigs	Estimated coverage (×)	Reads	Average raw read length	GC %	N50 (bp)	Collection site
*Lactiplantibacillus pentosus*										
7.2.15	JAVLAU000000000	SRR25916667	3,919,042	8	157.542	63,240	9,772	45.93	3,645,019	Mount Olive, NC
LA0445	JAHLEN000000000.2	SRR25916659	3,945,202	11	71.017	42,596	6,588	45.84	3,580,249	Mount Olive, NC
1.8.18	JAVLAN000000000	SRR25916663	3,897,544	15	108.48	47,598	8,910	45.82	2,062,758	Mount Olive, NC
1.8.9	JAVLAO000000000	SRR25916661	3,734,812	12	90.879	50,246	6,761	45.92	3,419,212	Mount Olive, NC
14.8.42	JAVGXB000000000	SRR25916660	4,050,922	16	62.214	32,012	7,882	45.78	789,853	Mount Olive, NC
7.8.46	JAVLAQ000000000	SRR25916658	3,966,095	12	106.639	46,231	9,160	45.73	2,973,648	Mount Olive, NC
7.8.2	CP134788-CP134793	SRR26196798	3,919,042	8	157.542	63,240	9,772	45.93	3,645,019	Chaska, MN
*Lactiplantibacillus plantarum*										
7.8.4	JAVLAR000000000	SRR25916657	3,490,038	15	44.239	23,524	6,609	44.23	2,712,281	Mount Olive, NC
*Lactococcus lactis*										
NCK401	CP137623	SRR26043614	2,551,975	1	75.178	25,782	7,450	34.86	2,551,975	Raleigh, NC
LA0312	CP134164	SRR25916659	2,563,933	1	172.327	49,924	8,857	34.86	2,563,933	Mount Olive, NC
*Pediococcus pentosaceus*										
LA0061	CP137627-CP137629	SRR26043613	1,945,268	3	250.134	51,502	9,455	37.19	1,847,864	Raleigh, NC
*Leuconostoc mesenteroides*										
FFL48	CP137625-CP137626	SRR26043612	1,816,909	2	252.299	53,393	8,589	38.02	1,799,974	Marshall, MI
*Companilactobacillus alimentarius*										
7.2.6	JAVKYT000000000	SRR25916668	2,665,037	10	192.679	56,034	9,171	35.5	1,641,583	Mount Olive, NC
*Levilactobacillus namurensis*										
1.2.9	JAVLAL000000000	SRR25916666	2,932,609	10	173.356	64,197	7,925	50.77	2,656,307	Mount Olive, NC
3.8.38	JAVLAM000000000	SRR25916665	2,983,047	14	152.717	57,348	7,938	50.68	2,566,380	Mount Olive, NC
90.8.26	CP134159-CP134163	SRR25916664	2,776,426	5	196.758	59,866	9,290	51.4	2,674,724	Mount Olive, NC
*Latilactobacillus zymae*										
90.8.36	JAVLAS000000000	SRR25916656	2,892,482	8	170.533	56,995	8,701	51.49	2,645,371	Mount Olive, NC
*Latilactobacillus curvatus*										
3.8.43	JAVLAK000000000	SRR25916651	2,056,476	4	237.549	62,061	7,860	42.03	1,616,338	Chaska, MN
*Lysinibacillus capsici*										
Lys1	CP137622	SRR26060012	4,682,737	1	94.303	49,304	8,969	37.58	4,682,737	Mount Olive, NC
Lys24	CP134502	SRR26060010	4,687,025	1	87.806	48,098	8,564	37.62	4,687,025	Mount Olive, NC
*Lysinibacillus louembei*										
Ll15	CP137624S	SRR26043611	3,960,254	1	93.807	45,092	8,245	38.53	3,960,254	Mount Olive, NC
*Streptococcus parasanguinis*										
30.8.10	CP134147-CP134148	SRR25916648	2,128,578	2	199.9238186	51,124	8,334	42.01	2,118,583	Mount Olive, NC
*Enterobacter cancerogenus*										
3.2.13	JAVLAJ000000000	SRR25916674	4,902,923	4	72.304	53,067	6,685	55.64	4,466,362	Chaska, MN
3.2.17	CP134403-CP134405	SRR25916652	4,882,814	3	131.134	68,845	9,309	55.63	4,668,754	Chaska, MN
*Enterobacter hormaechei*										
1.2.3	CP134406-CP134411	SRR25916671	3,862,785	6	121.153	51,423	9,112	46.03	3,635,834	Mount Olive, NC
3.2.8	JAVLAI000000000	SRR25916670	5,031,271	6	68.791	52,737	6,568	55.41	3,591,916	Mount Olive, NC
*Kluyvera cryocrescens*										
1.8.5	CP134165	SRR25916669	4,849,988	1	120.883	60,604	9,685	53.92	4,849,988	Mount Olive, NC
*Leclercia pneumoniae*										
1.2.7	CP134503-CP134506	SRR26196799	4,932,015	4	129.539	68,633	9,317	54.49	4,521,154	Mount Olive, NC
*Pantoea agglomerans*										
1.2.4	CP134149-CP134151	SRR25916650	4,870,469	3	125.758	69,211	8,857	55.14	4,084,927	Mount Olive, NC
*Pseudomonas putida*										
1.8.4	CP137621	SRR26043609	5,946,927	1	124.581	80,233	9,246	61.9	5,946,927	Chaska, MN
*Pseudomonas inefficax*										
7.8.24	CP134401	SRR25916649	5,927,592	1	113.28	73,885	9,099	62.78	5,927,592	Chaska, MN
*Erwinia toletana*										
1.2.20	CP134152-CP134153	SRR25916662	5,014,558	2	117.097	67,679	8,696	52.22	4,923,789	Chaska, MN
*Siccibacter colletis*										
3.2.4	CP134402	SRR25916654	4,158,854	1	135.985	67,089	8,438	57.41	4,158,854	Chaska, MN
*Hafnia alvei*										
3.8.4	CP134154-CP134158	SRR25916653	4,855,803	5	109.27	56,367	9,421	48.81	4,631,589	Chaska, MN
*Stenotrophomonas indicatrix*										
7.2.7	JAVYIJ000000000	SRR26060011	4,573,902	9	108.262	72,052	6,878	66.29	2,356,763	Chaska, MN
*Brucella pseudogrignonensis*										
1.2.1	JAVLAT000000000	SRR25916655	4,453,607	7	130.227	63,658	9,118	51.87	2,323,104	Chaska, MN

^*a*^ sites of sample collection are abbreviated as NC, MI, and MN for North Carolina, Michigan, and Minnesota, USA, respectively.

Pure cultures of lactic acid bacteria and bacilli were transferred from frozen stocks to Lactobacilli deMan, Rogosa, and Sharpe (MRS) broth, while proteobacteria were transferred to Brain Heart Infusion (BHI) broth and incubated statically and aerobically at 30°C prior to DNA extraction. The Promega Wizard Genomic DNA Extraction Kit (Madison, WI) was used to lyse cells and precipitate proteins. To produce a higher DNA yield than possible with the standard kit protocol, cell lysates were treated with 25:24:1 phenol:chloroform:isoamyl alcohol followed by ethanol precipitation (9).

Genomic DNA was sheared using Covaris G-tubes (Woburn, MA) targeting 10 kb fragments. Sheared DNA was prepared for PacBio sequencing using the SMRTbell Prep Kit 3.0 (PacBio, Menlo Park, CA). Samples were barcoded and libraries were size selected with AMpure PB beads (Pacific Biosciences, Menlo Park, CA) to remove fragments less than 3 kb. Sequencing was performed on a Sequel IIe System (Pacific Biosciences, Menlo Park, CA) using Binding Kit 3.2, Sequel II Sequencing Kit 2.0 and SMRTCell 8M. To target HiFi reads, the library was sequenced using a 30-hour movie time using Instrument Control Software Version 11. Raw subreads were converted to HiFi data by processing with CCS to call a single high-quality consensus sequence for each molecule, using a 99.5% consensus accuracy cutoff. *De novo* assembly was performed in BV-BRC v. 330.19a (10) via Unicycler version 0.4.8 (11) with a minimum contig cut-off of 300. Quality assessment of assemblies was performed with QUAST version 5.0.2 (12), SamTools version 13 (13), and Pilon version 1.23 (14). Closest reference genomes were identified by Mash/MinHash employing the PATRIC database (15). Upon submission to GenBank (Bioproject PRJNA674638), assemblies were reannotated using the NCBI Prokaryotic Genome Annotation Pipeline v. 6.5 (16). Default parameters for the software were used.

## Data Availability

Strain identification and accession numbers for each genome annotation and sequence read archive are included in Table 1.
